# Antifungal Compounds from Microbial Symbionts Associated with Aquatic Animals and Cellular Targets: A Review

**DOI:** 10.3390/pathogens12040617

**Published:** 2023-04-18

**Authors:** Madeleine Nina Love Ngo-Mback, Elisabeth Zeuko’o Menkem, Heather G. Marco

**Affiliations:** 1 Institute of Fisheries and Aquatic Science, University of Douala, Douala P.O. Box 2701, Cameroon; 2 Department of Biomedical Sciences, University of Buea, Buea P.O. Box 63, Cameroon; 3 Department of Biological Sciences, University of Cape Town, Private Bag X3, Rondebosch, Cape Town 7701, South Africa

**Keywords:** antifungal compounds, microbial symbionts, aquatic animals, cellular targets

## Abstract

Fungal infections continue to be a serious public health problem, leading to an estimated 1.6 million deaths annually. It remains a major cause of mortality for people with a weak or affected immune system, such as those suffering from cancer under aggressive chemotherapies. On the other hand, pathogenic fungi are counted among the most destructive factors affecting crops, causing a third of all food crop losses annually and critically affecting the worldwide economy and food security. However, the limited number currently available and the cytotoxicity of the conventional antifungal drugs, which are not yet properly diversified in terms of mode of action, in addition to resistance phenomena, make the search for new antifungals imperative to improve both human health and food protection. Symbiosis has been a crucial alternative for drug discovery, through which many antimicrobials have been discovered. This review highlights some antifungal models of a defensive symbiosis of microbial symbiont natural products derived from interacting with aquatic animals as one of the best opportunities. Some recorded compounds with supposed novel cell targets such as apoptosis could lead to the development of a multitherapy involving the mutual treatment of fungal infections and other metabolic diseases involving apoptosis in their pathogenesis pathways.

## 1. Introduction

Fungal infections are caused by pathogenic fungi that can be superficial at the level of the skin or biological mucosa, or they can be systemic, disseminated inside the whole body or on a particular inner organ, such as the lung, brain, heart, liver, and blood. These infections continue to be a serious public health problem, leading to an estimated 1.6 million deaths annually [1]. Fungal infections are dangerous and remain a major cause of mortality for people with a weak or affected immune system, such as those suffering from cancer under aggressive chemotherapies. Besides, the number of immunocompromised is growing due to an increase in the availability of hospital care, the introduction of suppressive immunotherapy, and the emerging immune diseases such as immunodeficiency viral infections, cancers, and organ transplantation that make these vulnerable populations exposed to such opportunistic fungal infections. On the other hand, pathogenic fungi are counted among the most destructive factors affecting crops, causing a third of all food crop losses annually, which critically affects the worldwide economy and food security. Aquaculture is also impaired by pathogenic fungi, which reduce fish production yield. According to the FAO’s report from 2009 to 2010 (https://www.fao.org accessed on 2 February 2023), fungal infections affect the most important crops in the world in order of occurrence: maize, potatoes, soybean, rice, and wheat. Because of these crop losses, 61% of the worldwide population will lack food [2,3]. However, the limited number currently available and the cytotoxicity of the conventional antifungal drugs, which are not yet properly diversified in terms of mode of action, in addition to resistance phenomena, make the research for new antifungals imperative to improve both human health and crop protection [4,5,6]. For many years, research carried out for drug discovery has involved a lot of natural and artificial sources. Among them, natural sources seemed to represent the most abundant and diverse place to find new bioactive molecules. In that way, symbiosis has been a crucial alternative for drug discovery, through which many antimicrobials have been discovered. Symbiosis can be mutualistic, commensal, or parasitic. Further, it can be optional when both organisms live independently or obligatory when these organisms need each other to survive. During that biological collaboration, a set of secondary metabolites is produced, playing an important role in the management of the interactions between the concerned organisms [7]. These metabolites could serve as a starting point for drug discovery and production.

The discovery of antimicrobials derived from microorganisms involved in symbiosis constitutes a field of research that is not widely explored. However, some bioactive molecules against pathogenic fungi have been isolated from bacteria on *Acromyrmex octospinosus* ants. Nystatin P1 is an antifungal compound produced by *Pseudonocardia* sp. through a nystatin-like biosynthetic gene cluster identified in the bacteria as symbionts on *A. octospinosus* [8]. Moreover, it was recently shown that candicidin and antimycins are also produced by *Streptomyces* sp. symbionts and exhibit antifungal activity against *Candida albicans* (*C. albicans*) and *Escovopsis* sp. [9,10]. Griseofulvin too (7-chloro-4,6,2′-trimethoxy-6′-methylgris-2′-en-3,4′-dione) is an antifungal mycotoxin isolated from *Penicillium* species. It has been widely used for the treatment of superficial dermatomycoses [11]. The mycotoxin was first isolated from a terrestrial fungus, the mold *Penicillium griseofulvum* [12]. The bioactive compound was also detected in the aquatic animal sponge *Axinella verrucosa* (*A. verrucosa*) through its fungal symbiont from the *Penicillium* genus. The ethyl acetate extract of the *Penicillium* yielded the production of griseofulvin [13].

This review highlights the involvement of microbial symbiont interactions with aquatic animals and the possibility of biomimicry of these interactions for the discovery of new bio antifungals with new cellular targets to better fight antimicrobial resistance and provides recommendations for future research concerning antifungal discovery. The next sections of this work will be focused on the explanations of why attention is being turned to microbial symbiosis with aquatic animals, in particular, some antifungal compounds isolated from such symbiosis together with their cellular targets and recommendations concerning future research studies.

## 2. A Microbial Symbiosis Approach Associated with Aquatic Animals for Antimicrobial Discovery

The current available antifungal drugs exhibit toxicity and resistance phenomena. Pathogenic fungi are eukaryotic cells, just as are human cells. So, they share the same targets, which do not ease the development of new drugs and lead to cytotoxicity. On the other hand, the limited available classes or cell targets of these antifungals promote the development of antibiotic resistance. Regarding the current and persistent problem in that research field, new antifungal molecules are now in the development process. However, a lot of these new molecules still target the same fungal cell sites [6], which predicts future resistance. So, to contribute to expanding the research on new compounds with new cell targets, aquatic environments with an emphasis on microbial symbiosis in aquatic animals are proposed here as potent bioresources.

Water as oceans is the most abundant molecule on the earth’s surface, occupying more than 70% of the surface of the planet with the highest worldwide biodiversity [14]. About 1.5 million species have already been discovered worldwide. Of those, 1.5 million, or 80%, are of terrestrial origin, whereas aquatic environments only account for 20% [15]. However, some scientists affirmed that the more complex or larger an ecosystem is, the greater its biodiversity. According to this approach, aquatic ecosystems, which are more abundant on the planet, may contain more new species than terrestrial surfaces. But the lowest percentage of the identified species in water environments could be justified by the fact that water ecosystems are underexplored, which opens new perspectives for the discovery of new animal organisms with potent microbial symbionts.

The investigation was focused on symbiosis associated with animals because Homo sapiens and animals are biologically closed [16,17]. Moreover, for in vivo tests using animal models, the human response is closely compared to one of those animals. It does not mean that they are identical. However, their defense systems against a toxic or a microbial aggressor sometimes involve the same biochemical pathways. Animal biology remains the focal point for studying humanity’s survival strategies [18]. Furthermore, this approach could well be applied to the protection of plants and foodstuffs because some pathogenic fungi affecting animals also cause diseases on plants and various foodstuffs, but above all, they all often share similar cell structures, which could represent common antifungal targets. This broadens the scope of research for antifungals against human, plant, and other food infections starting from the same bioresource represented by microbial symbioses of aquatic origin.

Aquatic animals use many features to defend themselves against a plethora of infectious diseases. These features include mucus, skin, and gills, which constitute protection toward their natural niches and against pathogenic agents. Mucus is found on the skin, gills, and intestines of organisms and contains antimicrobial agents such as proteins, lysozyme, immunoglobulin, enzymes, and lectins that could benefit human health research in clinical studies [19]. In that way, some experimental activities have shown the antimicrobial activities of the mucus extracts of *Maculabatis gerrardi* and *Pastinachus sephen* against *Candida tropicalis* (*C. tropicalis*), *Aspergillus niger* (*A. niger*), *Penicillium* sp., *Trichophyton mentagrophytes*, *Alternaria alternata (A. alternata)*, *C. albicans*, *Rhizopus* sp., *Mucor* sp., and *Trichophyton rubrum* [20]. A methanol extract of *Perna viridis* gills also exhibited potent antifungal activity against *A. niger* and *Mucor* sp. Moreover, in amphibians, some symbiotic skin bacteria located on the frog skin produce antifungal metabolites towards the cutaneous pathogen *Batrachochytrium dendrobatidis* (*B. dendrobatidis*), which has caused many amphibian population declines and extinctions [21]. On the other hand, some studies suggest that the population of microbial symbionts existing in mucus is responsible for the production of specialized metabolites involved in the management of host pathogen proliferation. In addition to mucus, other organs such as gills, skin, gut, and olfactory organs display a wide diversity of microorganisms living in symbiosis with aquatic animals, which are responsible for anti-infectious activity [22,23].

So, in the context of natural product chemistry, defensive symbioses can produce novel bioactive compounds to protect the host, leading to an interest in human health and crop protection [24]. Symbiosis interactions offer a biological platform for the exchange of bioactive compounds between the host and the microbial community. The naturally produced products act through different mechanisms. They could function as deterrents to predation, nutritional sources, immunomodulatory factors, genes transferred to the host, or antibiotics [25]. Our field of interest resides in their antibiotic function.

One of the best opportunities to exploit the chemical-mediated defensive symbiosis interaction between microorganisms and aquatic animals is to biomimic the mechanism of that defense against infectious agents. That is why investigating animal defense systems against fungal aggressors through biomimicry could shed light on new effective antifungal agents to treat fungal infections in humans, other animal species, and even crops.

## 3. Aquatic Animal-Microbial Symbionts Derived Antifungal Compounds and Their Targets

Some models of defensive symbiosis between microbial symbionts and natural products derived from their interactions with aquatic animals have been defined here. The natural products are grouped according to 7 cell targets (cellular enzymes, resistance factors, cell wall, cell multiplication and differentiation, plasma membrane, immunomodulation-apoptosis, and multicellular targets). Human fungal pathogenic fungi, *Aspergillus* sp.*, *Candida* albicans,* and *Trichophyton* sp., were susceptible to 1–3 µg/L concentrations of the bioactive compound. Concerning plant protection, inhibitory values were recorded up to 0.5 µg/L and 0.39 μmol/L, 25-fold stronger than that of the positive control, ketoconazole, for the inhibition of *Phytophthora capsici* (*P. capsici*) zoospore motility and *Pestalotia calabae* (*P. calabae*), respectively. The following Table 1 highlights the details of the bioactive compounds, their producing sources, their antifungal cellular targets, and the available chemical structure.

### 3.1. Compounds Acting on the Plasma Membrane

A total of five organic compounds were identified as acting on the plasma membrane: iturin, 3,5-dibromo-2-(3,5-dibromo-2-methoxyphenoxy) phenol, YM-202204, theonellamide F, and theopalauamide.

#### 3.1.1. Iturin

*A. aerophoba* is a sponge that contains a large number of bacteria. Antimicrobial activities of bacterial isolates from *A. aerophoba* were tested against many microbial pathogens. The results showed that *B. subtilis* strains A184, A190, and A202 exhibited strong activity against the fungus *C. albicans* [13,90]. According to some research results, the fungicidal activity in Bacillus is an indicator of the presence of lipopeptide from the iturin (compound **9**) class [90]. Compound **9** is included in the lipopeptide group, which acts as an immune stimulator in plants. Its main structure is heptapeptides linked to a β-amino fatty acid chain with a length of 14 to 17 carbons and a molecular mass of ~1.1 kDa [91]. The differences in heptapeptides show derivative compounds of iturin (e.g., bacillomycin, mycosubtilin) [44]. Their action mechanism involves the disturbance of the plasma membrane of the fungal target. The compound affects the morphology and membrane structure of yeast cells by increasing the electrical conductance of bimolecular lipid membranes and acting as a nontoxic and nonpyrogenic immunological adjuvant [43,46].

#### 3.1.2. 3,5-Dibromo-2-(3,5-dibromo-2-methoxyphenoxy)phenol

It has been proven that *Vibrio* sp. bacteria isolated from the sponge *Dysidea* sp. were able to biosynthesize 3,5-dibromo-2-(3′,5′-dibromo-2′-methoxyphenoxy) phenol (compound **10**). The compound was isolated from ethanol extracts of the sponge *Dysidea* sp. It belongs to the group of brominated diphenyl ethers, with a molecular mass of 531.82 g/mol [47]. Inhibitory parameters of compound **10** were determined against pathogenic fungi. *A. fumigatus*, *A. fumigatus, C. albicans,* and *C. tropicalis* were inhibited at 7.8 mg/mL. *A. flavus* and *C. glabrata* have shown susceptibility concentrations of 1.95 mg/mL and 15.2 mg/mL, respectively. Moreover, the compound exhibited fungicidal activity against *A. fumigatus* at 15.62 mg/mL and against *C. albicans* at 7.81 mg/mL. The investigation of a possible target revealed the disruption of the fungal cell membrane, expressed primarily in the leakage of potassium ions [48].

#### 3.1.3. YM-202204

YM-202204 (compound **11**) is an antifungal that also exhibits antibacterial properties. It was discovered in the culture broth of the marine fungus *Phoma* sp. Q60596. The structure is a pyrone determined by several spectroscopic experiments as a lactone compound appearing as a yellow syrup with a molecular mass of 646 g/mol [49]. Some researchers have shown that pyrenes could also be isolated from other fungi, such as *Fusarium* species [49,92]. *C. albicans*, *C. neoformans,* and *A. fumigatus* were highly susceptible to compound **11**. The latter inhibited glycosyl-phosphatidyl-inositol (GPI)-anchoring in yeast cell membranes [49].

#### 3.1.4. Theonellamide F

Marine sponges from the *Theonella* and *Discodermia* genera contain a reserve of bioactive metabolites. Theonellamide F (compound **12**) was isolated from alcohol and aqueous extracts of the marine sponge, genus *Theonella*. It is a dodecapeptide composed of L-Asn, L-*a*Thr, two residues of L-Ser, L-Phe, *b*Ala, (2S,3R)-3-hydroxyasparagine, (2S,4R)-Z-amino-4-hydroxyadipic acid, *r*-L-histidine-D-alanine, L-*p*-bromophenylalanine, and (3S,4S,5E,7E)-3-amino-4-hydroxy-6-methyl-8-*p*-bromo- phenyl)-5,7-octadienoic acid, with an unprecedented histidinoalanine bridge [50,93]. Compound **12** showed activity against several fungi, including *Candida* spp., *Trichophyton* spp., and *Aspergillus* spp., at active concentrations of 3.2–12 µg/mL. More recent biological studies using mutations/deletions of genes in the ergosterol biosynthetic pathway and calcein as a fluorescent dye have respectively shown that the yeast *Cerevisiae pombe* displayed high tolerances or slight resistances to compound **12** and a loss of membrane integrity when treated with the dye [52]. Theonellamides represent a new class of sterol-binding agents [51]. 

#### 3.1.5. Theopalauamide

*T. swinhoei* is a Palauan sponge that contains bacterial symbionts that produce theopalauamide (compound **13**). These bacterial symbionts are composed of *C. Entotheonella palauensis* from the d-subdivision of proteobacteria. Compound **13** is an antifungal, bicyclic glycopeptide presented in the form of a white powder with a molecular mass of 1746.9 g/mol [57]. Concerning its biological activity, yeast chemical-genomic approaches were used to determine the mode of action. The results of several research approaches have shown that theopalauamide represents a new class of sterol-binding compound. Biochemical experiments have specifically identified ergosterol as the primary target of theopalauamide [55,56].

### 3.2. Immunomodulation and Apoptosis

Mammals share with some pathogenic fungi two orthologous apoptosis genes: apoptosis-inducing factor (Aif) and endonuclease G (NuGl) that are responsible for programmed cell death (PCD), which represent challenging antifungal targets [94,95]. Two compounds (indole-3-carboxaldehyde and isatin) can modulate the immune system in order to stop the fungal infection and activate a cell-programmed death in the pathogen.

#### 3.2.1. Indole 3-carboxaldehyde

Microbial symbiosis relationships with animals are not strange. Surface bacteria from amphibians’ skin are beneficial to the host amphibian [96]. The fungus *J. lividum,* living at the skin surface of the red-backed salamander *P. cinereus* (characterized by its ability to exploit both aquatic and terrestrial habitats), synthesizes secondary metabolites that inhibit the pathogenic fungus *B. dendrobatidis,* which is a chytrid causing Chytridiomycosis. Indole-3-carboxaldehyde (compound **14**) is the heteroarenecarbaldehyde produced. The indole is a heteroarenecarbaldehyde, an indole alkaloid with 145.16 g/mol of molecular mass. The latter inhibits the pathogen’s growth at 68.9 μM. These results suggest that cutaneous symbiosis bacteria lead to amphibian resistance to fungal diseases. In addition, compound **14** also interferes with the growth of *C. albicans* by reducing vulvovagival candidiasis, as shown in an in vivo model study. The presence of compound **14** in the animal organism acts as immunomodulatory by stimulating the production of IL-22 via the aryl hydrocarbon receptor (AhR), promoting IL-18 expression, and providing protection against *Candida* infection [59].

#### 3.2.2. Isatin

*P. macrodactylus* is a shrimp that is highly resistant to a fungal infection at its embryonic stage. The pathogenic fungus that causes the infection is *L. callinectes,* which also affects many other crustaceans. A particular bacterium, *Alteromonas* sp., spreads at the external envelope of the crustacean embryos and produces 2,3-indolinedione, commonly called isatin (compound **15**). The compound inhibits the pathogenic fungus. Compound **15**, which is also found in plants and other animals, possesses an indole ring structure, which is common to many pharmaceuticals and heterocyclic natural products of biological interest [97]. If exposed to the fungus, bacteria-free embryos quickly die, whereas similar embryos reinoculated with the bacteria or treated only with compound **15** survive. So, the symbiotic *Alteromonas* sp. bacteria protect *P. macrodactylus* embryos against *L. callinectes* infection by releasing the antifungal compound **15** [62]. In addition, other scientists have revealed the susceptibility of *C. albicans*, *C. monosa*, *C. glabrata*, *T. longifusus*, *M. canis*, and *A. flavus* to indole (0.25–1 mg/mL) [60,61]. While studying the mechanism of action of compound **15**, closely related compounds have shown apoptotic effects on *C. albicans* at sub-inhibitory concentrations, suggesting targeting apoptosis could be considered as an alternative to be explored for antifungal drug discovery [63].

### 3.3. Cell Differentiation and Multiplication

Griseofulvine, majusculamide C, and surfactin are the three organic compounds that act as chemical interference in the multiplication and differentiation of pathogenic fungi by blocking mitosis and the genetic material of the pathogens.

#### 3.3.1. Griseofulvin

The *Penicillium* fungus was isolated from the sponge *A. verrucosa* in the Mediterranean Sea. The microorganism *Penicillium* sp. has been submitted to an ethyl acetate maceration to obtain solvent extracts containing griseofulvin (compound **6**) [13]. Compound **6** or 7-chloro-4,6,2′-trimethoxy-6′- methylgris-2′-en-3,4′-dione (352.8 g/mol) is a mycotoxin produced by various species of *Penicillium* [98]. Compound **6** disrupts the mitotic spindle by interacting with the polymerized microtubules, leading to the production of multinucleate fungal cells and stopping cell division at metaphase. The inhibition of nucleic acid synthesis and the formation of hyphal cell wall material also may be involved. The result is distortion, irregular swelling, and spiral curling of the hyphae. The bioactive molecule is fungistatic in mature dermatophytic cells and fungicidal in immature ones [99]. The isolated compound is a widely used antifungal drug for the treatment of superficial dermatomycoses. However, because it is carcinogenic and teratogenic in animal models, there is considerable concern regarding its clinical application. Further, it produces numerical chromosome aberrations in human lymphocytes and cell lines. There are conflicting reports on the ability of compound **6** to induce structural chromosomal aberrations. However, compound **6** induces micronucleus formation both in isolated peripheral lymphocytes and lymphocytes from whole blood cultures [11]. 

#### 3.3.2. Majusculamide C

A cyclic depsipeptide, majusculamide C (compound **7**), was isolated from the sponge *P. trachys* collected at the Enewetak Atoll (Marshall Island, Pacific Ocean). It was originally isolated from the toxic blue-green alga *Lyngbya majuscula* obtained from the same site. Compound **7** exhibited antifungal activity against pathogens of commercially important plants [13,38]. The compound is active against several plant pathogenic fungi such as *R. solani*, *P. aphanidermatum*, *A. euteiches,* and *P. infestans* at concentrations of, respectively, 4 µM, <1 µM, 2 µM, and 1 µM [40]. The cyclic depsipeptide acts as a microfilament-depolymerizing agent that shows potent fungicidal activity and may have been used in the treatment of resistant fungi-inducing diseases of domestic plants and crops [41].

#### 3.3.3. Surfactin

The microflora of *A. aerophoba* constituted by bacteria *B. subtilis* A190 *B. subtilis* A184 was responsible for surfactin class secondary metabolites production [13]. Surfactin (compound **8**), with a molecular weight of 1036.3 g/mol, was thus characterized as a lipopeptide composed of a heptapeptide with the following sequence: L-Glu1-L-Leu2-D-Leu3-L-Val4-L-Asp5-D-Leu6-L-Leu7, forming a lactone ring structure with a β-hydroxy fatty acid chain. Bearing both a hydrophilic peptide portion and a lipophilic fatty acid chain, compound **8** is amphiphilic, leading to exceptional biosurfactant activities and diverse biological activities [100]. The surfactin group has two polar amino acid residues, such as Glu and Asp, and it has been concluded that they bind with DNA via hydrogen bonds. The compound affected *F. moniliforme* growth by causing morphological changes in hyphae, suggesting that it markedly contributed to inhibiting fungal growth. DNA binding results indicated that the lipopeptide negatively influenced the maintenance of DNA integrity by binding to *F. moniliforme* DNA, which might in turn genetically affect DNA function for *F. moniliforme* growth [42].

### 3.4. Cellular Enzymes

Brefeldin A and roridin A were found to be cellular enzyme inhibitors. They inhibit acid phosphatase and dehydrogenase activity, applying their functions to the secretory pathways and the respiratory chain, respectively.

#### 3.4.1. Brefeldin A

*Penicillium* species isolated from *Annella* sea fan yielded Brefeldin A (compound **1**). The compound (C_16_H_24_O_4_) is a macrolide with a molecular mass of 280.36 g/mol and possesses antibiotic properties [26,27]. The pathogenic fungus *M. gypseum* SH-MU-4 has shown a high susceptibility to compound **1**, exhibiting a minimum inhibitory concentration value of 228.57 mM [101]. The study of the mode of action has revealed a dose-dependent inhibition of the cell-surface enzyme acid phosphatase (APase) in the periplasm of *C. albicans,* leading to intracellular accumulations of enzyme protein. Cells grown in the presence of compound **1** became denser than those grown in the absence of the active compound. It has been concluded that fungal cell-surface growth was also blocked by treatments containing compound **18**. The APase that was accumulated intracellularly migrated faster on SDS-PAGE, suggesting less N-linked glycosylation compared with the mature periplasmic APase produced in the absence of BFA. Pulse-chase experiments and gel-filtration of oligosaccharides released by Endo H treatment suggested that the core-glycosylated precursor form of APase accumulated in the presence of BFA [27].

#### 3.4.2. Roridin A

Roridin A (compound **2**) from *Myrotheeium roridum* and *Fusarium* sp. is a sesquiterpene mycotoxin (12, 13-epoxy-trichothec-9-ene moiety) developed by these microorganisms with a molecular mass of 532.6 g/mol [102,103]. To obtain the compound, organic solvent extraction is used by macerating *Myrothecium* sp. inside ethyl acetate-ethanol (EtOAc). The producing symbiont is isolated from the marine sponge *Axinella* sp. The EtOAc symbiont extract has inhibited the growth of *S. sclerotiorum*, *S. cerevisiae,* and *M. grisea*. After separation by silica gel column fractionation and high-performance liquid chromatography (HLPC), roridin A and trichothecenes have been purified. The inhibitory concentrations of the compounds revealed MIC values close to those of fluconazole [29]. The bioactive agent showed antifungal activity against *S. cerevisiae*, *M. grisea,* and *S. sclerotiorum* with minimum inhibitory concentrations (MIC) of 31.25, 125, and 31.25 μg/mL, respectively. In vitro antifungal tests showed that the purified fractions were active against *A. niger*, *T. rubrum,* and *C. albicans* with MICs of 31.25, 62.5, and 125 μg/mL, respectively [30]. Compound **2** was able to inhibit the dehydrogenase activity of *S. cerevisiae* by up to 1 µg/mL. Furthermore, preliminary attention to the structure–fungitoxicity relationship of roridin A, whose MICs are comparable to ketoconazole, allowed a temporary observation that the presence of a hydroxyl group at C-13, as well as the ether bondage between C-5 and C-13, could increase almost equally the fungicidal action against *A. niger* and *T. rubrum* [30]. 

### 3.5. Resistance Factors

Multidrug resistance factors evolve from drug efficacy failure, which is characterized by the development of tolerance to a range of drugs. There are also natural resistance factors, such as the development of spores when the fungus enters the nutritive restriction stage of its life. Modiolide A from *Paraphaeosphaeria* sp. and Fumiquinazoline A (compound **4**) from *A. fumigatus* were able to thwart fungal resistance.

#### 3.5.1. Modiolide A

Modiolide A (compound **3**) is produced by a fungus, *Paraphaeosphaeria* sp. (N-119), isolated from a marine horse mussel. The organic natural product appears as a colorless oil with a molecular mass of 198.22 g/mol and a chemical structure determined using spectroscopy. Compound **3** exhibited a wide antimicrobial potential against *N. crassa,* a pathogenic fungus, with a MIC value of 3 µg/mL, and against the bacteria *Micrococcus luteus* with an inhibition concentration of up to 16.7 µg/mL as MIC [32]. Moreover, it impairs *P. capsici* by destroying 100% of its zoospore motility within 30 min at a concentration of 0.5 μg/mL. Its zoosporicidal potency is probably due to the presence of epoxide-containing cyclic lactones, which may be responsible for the inhibitory and zoosporicidal activities [104]. These spores, which can locate the host using their infectious propagules and accumulate at the sites of the infection through particular chemical signals, highly contribute to the pathogenic success of the *Phytophthora* genus. Moreover, any impairment of the spore motility greatly reduces its pathogenesis [105,106]. 

#### 3.5.2. Fumiquinazoline

Fumiquinazoline A (compound **21**; 445.5 g/mol) originated from the pathogenic fungus *A. fumigatus,* located at the gastrointestinal tract as endosymbionts of the fish *Pseudolabrus japonicas*. The mycelia of the fungus were cultured, and the compound was extracted. It was shown that compound **21** possesses cytotoxic activity against P388 lymphocytic leukemia cells [35]. The natural product was also capable of expressing antifungal activities against phytopathogenic fungi such as *B. cinerea*, *A. solani*, *A. alternata* (Fries) Keissler, *C. gloeosporioides*, *F. solani*, *F. oxysporum* f. sp. *Niveum*, *F. oxysporum* f. sp. *Vasinfectum,* and *Gibberella saubinettii* with MIC values ranging from 12.5–50 μg/mL. It is worth pointing out that the activity was more pronounced toward *B. cinerea*, *A. solani*, *A. alternata*, and *G. saubinettii* than toward *Fusarium* species. In addition, compound **21** highlighted an antifeedant activity with a moderate antifeedant index (AFI) of 45%. Moreover, fumiquinazoline A showed moderate activity with an AFI of 45.0%. However, when structural modifications occur on the active compound by inserting one hydroxyl group into C-3 to produce 3-hydroxyfumiquinazoline A, the antifeedant activity has considerably decreased, up to an AFI of 7.5 [34]. The structures of fumiquinazolines are quinazolinones fused with a simple piperazine ring system or more complex spiro moieties. The fused rings, the basic amine, and the rich stereochemistry are structural features useful for structure-activity relationship studies [36].

### 3.6. Cell Wall

The fungal cell wall is the first point of contact that initiates infection. The synthesis of cellular fungal components and the surface proteins represent capital targets to develop more efficient therapeutics or vaccines [107].

#### Chitinase 34 kDa

Chitinase 34 kDa (compound **5**) is an enzyme extracted from the bacterial strain DA11 identified as *Streptomyces* sp. by 16S rDNA sequencing (GenBank accession no. DQ180128). The bacterial strain grows as a symbiont on the sponge *C. austrialiensis* [37]. A volume of 20 μL of the compound has been tested for its eventual antifungal activity against *A. niger* and *C. albicans*. After 120 h of incubation, an inhibition zone as a circle around compound **5** containing discs was identified, characterizing the inhibition surface of the fungus growth. The antifungal inhibition diameters were 10.98 ± 0.49 mm for *A. niger* and 10.48 ± 0.45 mm for *C. albicans*. The obtained results demonstrate the antifungal activity of the purified chitinase, which acts by degrading chitin from the cell wall. Potential uses of chitinases as biocontrol agents have been reported [108]. Chitinases from *S. griseus* have also shown antifungal activity against *Aspergillus* sp., *Phycomyces blakesleeanus*, *Trichoderma reesei*, *A. nidulans*, *B. cinerea*, *Fusarium culmorum*, *Gulgnardia bidwellii,* and *Sclerotia sclerotiorum*. The enzyme remains active at 85% within temperatures ranging from 30 °C to 45 °C. These results were comparable to those obtained from a chitinase isolated from a novel marine sediment *Streptomyces* strain, which exhibited activity at an interval temperature of 20 °C to 65 °C. For this last example, the best activity was noticed between 45 °C and 50 °C [109]. Moreover, it is also known that pH influences ionizations at the catalytic site. In that way, for some other *Streptomyces* species, the optimum chitinase activity was found to be included within a pH interval of 3.3 to 7.5 [110]. So, enzymatic functional features show that the extracted marine chitinase can be active under basic conditions because of its high pH tolerance and a maximum salinity tolerance of 45 g‰ psu. The latter justifies one of the characteristics of marine microorganisms, which is to tolerate salt in their living environment [111].

### 3.7. Compounds with Unknown Targets

The remaining compounds **18**–**48**, tambjamine C, tambjamine E, roridin D, violacein, tambjamine F, theonegramide G, theonegramide, tambjamine H, tambjamine I, tambjamine J, LL-Z1640-2, 1-hydroxy-6-methyl-8-(hydroxymethyl)xanthone, xestodecalactone B, peniciadametizine A, caerulomycin A, ND, 87.12 kDa active protein, 1-hydroxy-6-methyl-8-(hydroxylmethyl)xanthone, modiolide B, (3*S*)-(3,5-dihydroxyphenyl)butan-2-one, (3*R*,4*S*)-hydroxymellein, 3*R*)-6-methoxy-7-chloromellein, (3*R*)-6-methoxymellein, 4,8-dihydroxy-3,4-dihydro-2*H*-naphthalen-1-one, (3*R*,4*R*)-hydroxymellein, (*R*)-mellein, seragikinone A, microsphaeropsin, (3*S*)-(3,5-dihydroxyphenyl)butan-2-one, xestolactone B, and resorcylic acid lactones “zeaenol”, were recorded as derivating from microbial symbionts associated with aquatic animals. However, their antifungal targets were still not properly determined. Among these compounds, tambjamines (F, G, H–J) (compounds **22**, **23**, **25**–**27**) were obtained from *P. tunicata* on bryozoans and some other marine animals, showing antifungal activity against *M. furfur* (0.1–1 mg/mL) and *C. albicans* (1 mg/mL). LL-Z1640-2 (compound **28**) has exhibited an inhibiting potential at a MIC = 0.39 μmol/L, 25-fold stronger than that of the positive control ketoconazole against the fungal pathogen *P. calabae.* The compound also impaired the growth of *P. viticola* and *P. infestans*. Peniciadametizine A (compound **31**), which originated from the *P. adametzioides* AS-53a fungus found on an unidentified marine sponge, was able to inhibit *A. brassicae* (MIC: 4.0 µg/mL). In general, the antifungal mechanism of these compounds remains undefined, opening new investigative opportunities for the identification of their cell targets to eventually find new antifungal mechanisms of action [13,24,32,71,74,75,76,77,79,80,81,82,83,84,85,86,87,88,89,112,113].

## 4. Relevant Information for Future Research

Six main classes of antifungal drugs are currently available for the treatment of fungal infections. They include polyenes that act on membranes by binding ergosterol and azoles that lead to the accumulation of 14-methyl-sterol. Echinochandines target β-1,3-glucane on the cell wall, allylamines inhibit epoxydase, and griseofulvine acts on microtubule polymerization [114]. Some of the following identified compounds in the study highlighted novel insights into antifungal activity targets. In the study, three new targets were suggested. Fungal cellular enzymes such as acid phosphatase, GTPase, and dehydrogenase are targeted by Brefeldin A and Roridin A (compounds **1** and **2**). Moreover*,* modiolide A (compound **3**) acting on resistance factors, such as the fungal spore motility of *P. capsici* zoospores [32], constitutes a leading opportunity for developing therapeutics to specifically inhibit resistance factors. Finally*,* immunomodulation and apoptosis pathways were impaired by indole-3-carboxaldehyde and isatin (compounds **14** and **15**), which targeted immune cells as an immunomodulator in the vulvocandidiasis model and as an apoptosis enhancer on *Candida* and *Aspergillus* microorganisms. To the best of our knowledge, there are not yet conventional antifungal drugs targeting these three targets: cellular enzymes, resistance factors, and the immune/apoptotic pathway. However, the challenge behind this resides in the fact that these fungal eukaryotic cells sometimes share the same cellular features as human eukaryotic cells, such as cellular enzymes, the immune system, and apoptosis. For example, apoptosis biochemical mechanisms are also integrated into human cells’ metabolic pathways [95,96,115]. Unless a specific antifungal therapy is only for cancer, people acting on apoptosis should be considered. So, deep investigations through preclinical and clinical trials involving toxicity studies may produce effective results in the development of new antifungal drugs with novel cell targets from aquatic sources. Actually, from these reports, spore motility seems to represent the best suggested novel antifungal target to investigate. Even the combination of these compounds with different and new cell targets may lead to promising effects. 

The multiple advantages of the combination approach have already been shown [116,117,118]. This approach exploits various beneficial characteristics associated with each combination product for the improvement of the effectiveness, the reduction of toxicity, the reduction of the doses to be administered, and the reduction of the features associated with the development of drug resistance [119]. In addition, this approach would involve a greater diversity of secondary metabolites in antimicrobial action. Combinations could therefore possibly target several metabolic pathways at once, thereby enhancing the desired therapeutic effect, most likely through synergistic interactions [120]. That is also the case with diacetylphloroglucinol (compound **16**), which acts at the same time on the mitochondrial membrane and causes hyphal disorganization. In addition, tyrosol (compound **17**) affects cell membrane permeability and inhibits the biofilm on *Candida* at the same time to produce the inhibition activity. 

## 5. Conclusions

The present study gives insights into how to learn from the microbial symbiosis associated with aquatic animals and develop mimicry strategies for the development of infectious disease treatments, particularly in the field of antifungals. Natural products derived from such symbiotic interactions may be mimicked, or the physical behavior of some fungi to protect the host could also be integrated into the antimicrobial development strategies. One example of chemically mediated defense from a microbial symbiont to protect its host is the case of *P. macrodactylus* embryos (crustacean), which defend themselves through the action of a symbiont bacteria, *Alteromonas* sp., to fight the fungal infection caused by *L. callinectes* on its embryos [62] by producing isatin, an antimicrobial agent to impair the progression of the infection, where close derivative isatin compounds act by activating the pathogen apoptosis pathway. Thus, three main targets to investigate in preclinical and clinical trials have been identified as potent antifungal cell targets as well as antifungal agents in cancer therapies. Besides, it is worth noting the presence of the identified antifungal targets here in this study as acid phosphatases, GTPases, and apoptosis, which are also involved in some cancer pathologies. Some of the mentioned compounds with apoptotic effects could also be involved in the drug discovery process of metabolic diseases because the same pathogenesis pathway as apoptosis activation is highlighted in cancer (compounds **1**, **2**, **7**, **15**, **21**, **26**, and **27**).

## Figures and Tables

**Table 1:** Antifungal compounds from M.O symbiont on marine animals.

Targets	Nb	Compounds	Producing Sources		Inhibited Fungi	* Chemical Formula	References
			M.O. Symbiont	Aquatic Animals			
**Cellular enzymes**	**1**	Brefeldin A	*Penicillium* sp.	Annelle sea fan	*Microsporum gypseum (M. gypseum) Candida* sp.	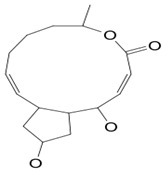	[26,27,28]
	**2**	Roridin A	*Myrothecium* sp.	*Sponge Axinelle* sp.	*Saccharomyces cerevisiae* (*S. cerevisiae*) *Magnaporthe grisea* (*M. grisea*) *Sclerotinia sclerotiorum (S. sclerotiorum)*	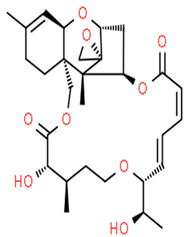	[29,30,31]
**Resistance factors**	**3**	Modiolide A	*Paraphaeosphaeria* sp.	*Modiolus auriculatus* (*M. auriculatus*) *P. capsici*	*Neurospora* *crassa (N. crassa)* *P. capsici*	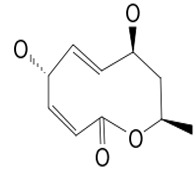	[32,33]
	**4**	Fumiquinazoline A	*Aspergillus fumigatus (A. fumigatus)*	*Pseudolabrus japonicus*	*Botrytis cinerea (B. cinerea)*, *Alternaria solani (A. solani)*; *(A. alternata)*, *Colletotrichum gloeosporioides (C. gloeosporioides)*, *Fusarium solani (F. solani)*, *Fusarium oxysporum (F. oxysporum)*, *Gibberella saubinettii*	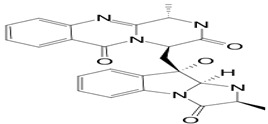 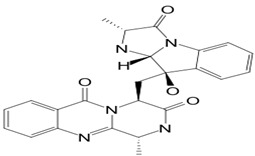	[34,35,36]
**Cell wall**	**5**	Chitinase 34 kDa	*Streptomyces* sp. DA11	Sponge *Craniella australiensis*	*A. niger* *C. albicans*	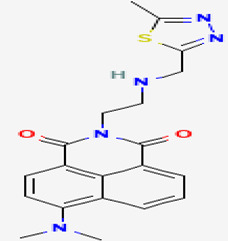	[37]
**Cell differentiation and multiplication**	**6**	Griseofulvin	*Penicillium* sp. (Ascomycota (fungus))	*A. verrucosa*	Dermatophytes	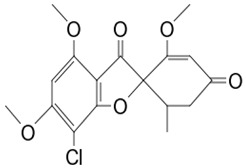	[11,13]
	**7**	Majusculamide C	*Lyngbya majuscule* *(a cyanobacteria)*	*Ptilocaulis trachys (P. trachys)*	*Rhizoctonia solani (R. solani)* *Pythium aphanidermatu (P. aphanidermatum)* *Aphanomyces euteiches (A.euteiches)* *Phytophthora infestans (P.infestans)*	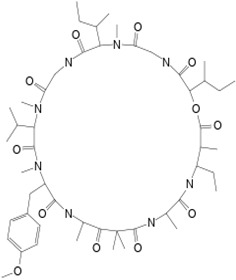	[13,38,39,40,41]
	**8**	Surfactin	*Bacillus subtilis* A190 (*B. subtilis*) *B. subtilis* A184	*Aplysina aerophoba (A. aerophoba)*	Antifungal *Fusarium moniliforme (F. moniliforme)*	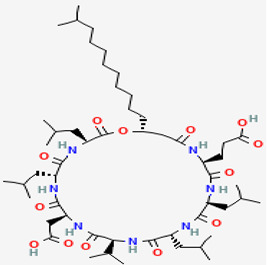	[13,42]
**Plasma membrane**	**9**	Iturin	*B. subtilis* A202 *B. subtilis* A184	*A. aerophoba*	*Fusarium* sp. *Penicellium* sp. *Monilinia* sp. *R. solani*	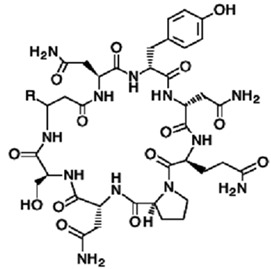	[13,43,44,45,46]
	**10**	3,5-dibromo-2-(3,5-dibromo-2-methoxyphenoxy)phenol	*Vibro* sp.	*Dysidea* sp. *Dysidea herbacea*	*A. fumigatus* *Aspergillus flavus (A. flavus)* *A. niger* *C. tropicalis* *C. albicans* *Candida glabrata (C. glabrata)*	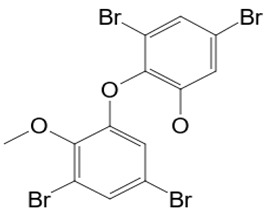	[35,47,48]
	**11**	YM-202204	*Phoma* sp. Q60596	sponge *Halichondria japonica*	*C. albicans* *Cryptococcus neoformans (C. neoformans)* *A. fumigatus*	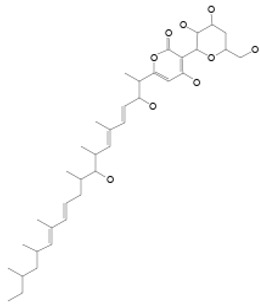	[49]
	**12**	Theonellamide F	-	*Theonella* sp.	*Candida* sp. *Trichophyton* sp. *Aspergillus* sp.	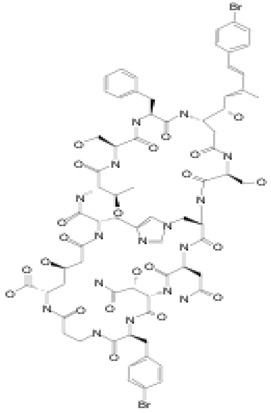	[50,51,52]
	**13**	Theopalauamide	*Candidatus Entotheonella palauensis*(*C. Entotheonella*)	*Theonella swinhoei (T. swinhoei)*	Fungi	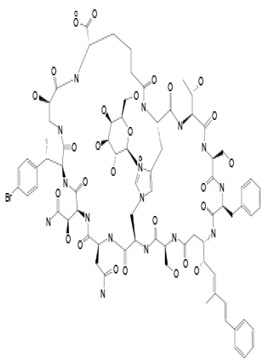	[53,54,55,56,57]
**Immunomodulatio n and apoptosis**	**14**	Indole 3-carboxaldehyde	*Janthinobacterium lividum (J. lividum)*	*Plethodon cinereus (P. cinereus)*	*B. dendrobatidis* *C. albicans*	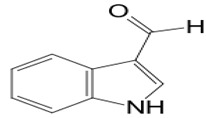	[58,59]
	**15**	Isatin	*Alteromonas* sp. (bacterium)	*Palaemon macrodactylus* (*P. macrodactylus) embryos*	*Lagenidium callinectes (L. callinectes)* *C. albicans* *Candida monosa (C. monosa)* *C. glabrata* *Tricophyton longifusus(T. longifusus)* *Microsporum canis (M. canis)* *A. flavus*	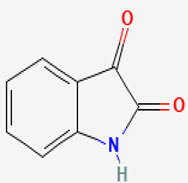	[60,61,62,63]
**Multicellular targets**	**16**	Diacetylphloroglucinol	*Lysobacter gummosus*	*P. cinereus*	*B. dendrobatidis* *Pythium ultimum* var. *sporangiiferum* *S. cereviaseae*	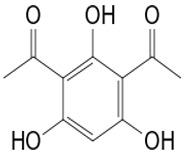	[64,65,66]
	**17**	Tyrosol	Bacterium SGT-76	*Homarus americanus* embryos	*L. callinectes* *C. glabrata* *Coccidioides posadasii* *Histoplasma capsulatum*	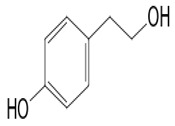	[67,68,69,70]
**Unknown targets**	**18**	Tambjamine C	*Pseudoalteromonas tunicata (P. tunicata)*	Bryozoans and some other marine animals	*Mallassezia furfur (M. furfur*) *C. albicans*	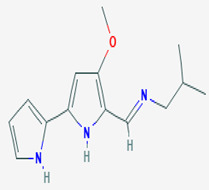	[24,71]
	**19**	Tambjamine E	*P. tunicata*	Bryozoans and some other marine animals	*M. furfur* *C. albicans*	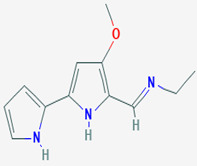	[24,71]
	**20**	Roridin D	*Myrothecium* sp.	*Sponge Axinelle* sp.	*S. cerevisiae* *M. grisea* *S. sclerotiorum*	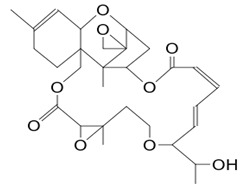	[29,30]
	**21**	Violacein	*J. lividum*	*P. cinereus*	*B. dendrobatidis*	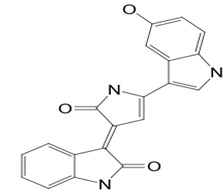	[58,72,73]
	**22**	Tambjamine F	*P. tunicata*	Bryozoans and some other marine animals	*M. furfur* *C. albicans*	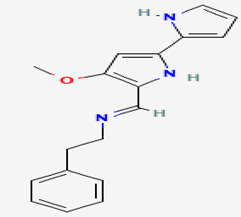	[24,71]
	**23**	Tambjamine G	*P. tunicata*	Bryozoans and some other marine animals	*M. furfur* *C. albicans*	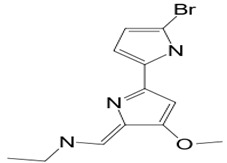	[24,71]
	**24**	Theonegramide	*C. Entotheonella palauenis* (δ- Proteobacteria)	*T. swinhoei*	*C. albicans (ATCC 32354)*	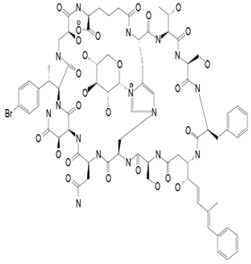	[13,74,75]
	**2*5***	Tambjamine H	*P. tunicata*	Bryozoans and some other marine animals	*M. furfur* *C. albicans*	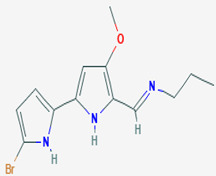	[24,71]
	**26**	Tambjamine I	*P. tunicata*	Bryozoans and some other marine animals	*M. furfur* *C. albicans*	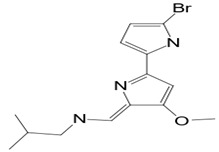	[24,71]
	**27**	Tambjamine J	*P. tunicata*	Bryozoans and some other marine animals	*M. furfur* *C. albicans*	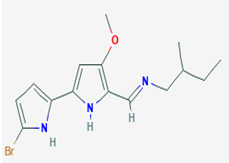	[24,71]
	**28**	LL-Z1640-2	*Cochliobolus lunatus (C. lunatus)*	Sea Anemone	*P. calabae*, *Plasmopara viticola (P. viticola) P. infestans*	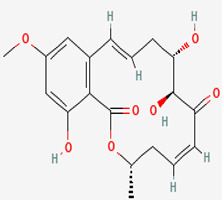	[76,77] Détail activités y est
	**29**	1-hydroxy-6-methyl-8-(hydroxymethyl)xanthone	*Ulocladium botrytis*	*Callyspongia vaginalis (C. vaginalis)*	Fungi	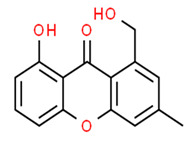	[78,79]
	**30**	Xestodecalactone B	*Penicillium* cf. montanense (P. montanense)	*Xestospongia exigua*	*C. albicans*	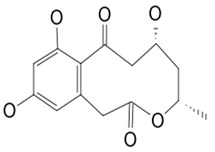	[13,80]
	**31**	Peniciadametizine A	*Penicillium adametzioides (P. adametzioides) AS-53*	An unidentified marine sponge	*Alternaria brassicae* (A. *brassicae*)	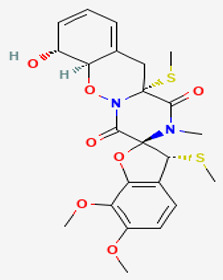	[81,82]
	**32**	Caerulomycin A	*Actinoalloteichus* sp.	A marine invertebrate	*Candida* sp.	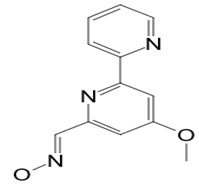	[81,83]
	**33**	Unidentified compound	*F. oxysporum* DLFP2008005	*Hymeniacidon* *perlevis*	Fungi	/	[13,84]
	**34**	87.12 KDa active Protein	*Nocardiopsis* *dassonvillei* MAD08	Sponge *Dendrilla nigra*	*Candida* sp.	/	[85]
	**3*5***	1-hydroxy-6-methyl-8-(hydroxylmethyl)xanthone	*Ulocladium botrylis*	Sponge *C. vaginalis*	*C. albicans*	/	[79]
	**36**	Modiolide B	*Paraphaeosphaeria* sp.	*M. auriculatus*	*N.* *crassa*	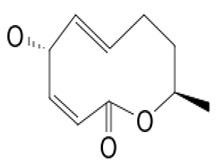	[32,33]
	**37**	(3*S*)-(3,5-dihydroxyphenyl)butan-2-one	*Coniothyrium* sp.	*Ectyplasia perox (E. perox)*	*Ustilago violacea (U. violacea)* *Mycotypha microspora*	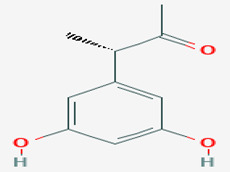	[78]
	**38**	(3*R*,4*S*)- Hydroxymellein	*Microsphaeropsis* sp.	*Myxilla incrustans (M. incrustans)*	*U. violacea*	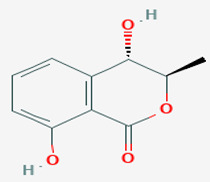	[78]
	**39**	3*R*)-6-methoxy-7-chloromellein	*Coniothyrium* sp.	*E. perox*	*Eurotium repens* *(E. repens)*	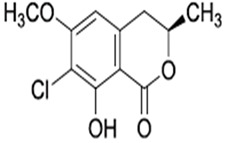	[78,86]
	**40**	(3*R*)-6-methoxymellein	*Coniothyrium* sp.	*E.perox*	*E. repens*	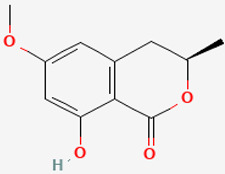	[78]
	**41**	4,8-dihydroxy-3,4-dihydro-2*H*-naphthalen-1-one	*Microsphaeropsis* sp.	*M. incrustans*	*E. repens* *U. violacea*	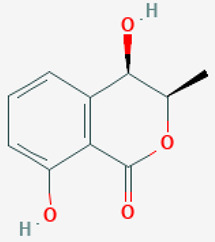	[78]
	**42**	(3*R*,4*R*)-hydroxymellein	*Microsphaeropsis* sp.	*M. incrustans*	*E. repens* *U. violacea*	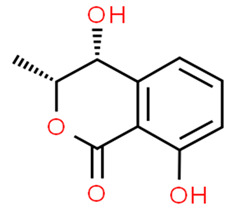	[78]
	**43**	(*R*)-mellein	*Microsphaeropsis* sp.	*M. incrustans*	*E. repens*	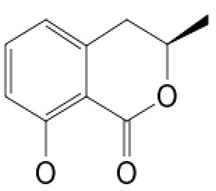	[78]
	**44**	Seragikinone A	Unidentified fungus	*Ceratodictyon spongiosum*	*C. albicans*	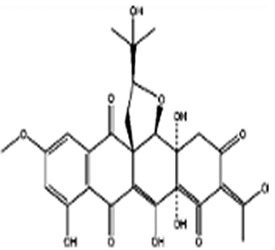	[26,33,87,88]
	**4*5***	Microsphaeropsin	*Microsphaeropsis* sp.	*M. incrustans*	*E. repens* *U. violacea*	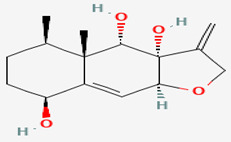 microsphaeropsin A 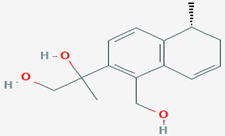 microsphaeropsin B	[33,78]
	**46**	(3*S*)-(3,5-dihydroxyphenyl)butan-2-one	*Coniothyrium* sp.	*E. perox*	*U. violacea* *Mycotypha microspora*	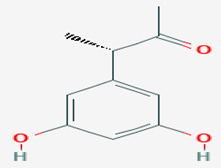	[78]
	**47**	Xestolactone B	*P.* cf. *montanense*	*Xestospongia exigua*	*C. albicans*	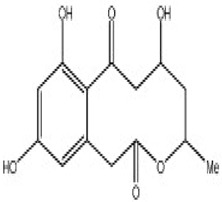	[26,33,80]
	**48**	Resorcylic acid lactones “zeaenol”	*C. lunatus*	*Palythoa haddoni*	*P. viticola,* *P.infestans*	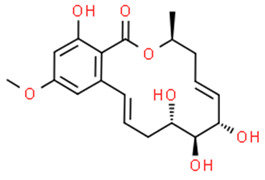	[76,89]

* Chemical structures of compounds were obtained from spectrabase, pubchem and chemspider https://spectrabase.com; https://pubchem.ncbi.nlm.nih.gov; http://www.chemspider.com/ (accessed on 5 January 2023).

## Data Availability

All studies from which data are presented are cited and referenced in the manuscript.
